# Crystal structure of 2-(4-*tert*-butyl­phen­yl)-3-hydroxy-4*H*-chromen-4-one

**DOI:** 10.1107/S2056989015011482

**Published:** 2015-06-24

**Authors:** Fuka Narita, Akihiro Takura, Takashi Fujihara

**Affiliations:** a Department of Chemistry, Graduate School of Science and Engineering, Saitama University, Shimo-Okubo 255, Sakura-ku, Saitama 338-8570, Japan; b Comprehensive Analysis Center for Science, Saitama University, Shimo-Okubo 255, Sakura-ku, Saitama 338-8570, Japan

**Keywords:** crystal structure, flavonol, hydrogen bonding, fluorescent material

## Abstract

Yellow–green fluorescent crystals of the title compound, C_19_H_18_O_3_, were obtained by the reaction of hy­droxy­aceto­phenone and 4-*tert*-butyl­benzaldehyde with hydrogen peroxide as oxidant. The plane of the benzene ring is slightly twisted to the mean plane of the 4*H*-chromene-4-one moiety (r.m.s. deviation = 0.0191 Å) by 10.53 (8)°. In the crystal, mol­ecules are linked by pairs of O—H⋯O hydrogen bonds, forming inversion dimers with an *R*_2_^2^(10) ring motif. The dimers are linked *via* C—H⋯π inter­actions, forming sheets parallel to (10-1).

## Abstract 2

The title compound is relatively planar with the benzene ring being only slightly twisted with respect to the mean plane of the 4*H*-chromene-4-one moiety (r.m.s. deviation = 0.0191 Å) by 10.53 (8)°. In the crystal, mol­ecules are linked by pairs of O—H⋯O hydrogen bonds, forming inversion dimers with an 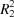
(10) ring motif.

## Chemical context   

The flavonol 3-hy­droxy-2-phenyl-4*H*-chromen-4-one (com­mon name: 3-hy­droxy­flavone) and its derivatives are present in a wide variety of plants as phytochemical compounds (Havsteen, 1983 ▸; Aherne & O’Brien, 2002 ▸). They have been investigated for many years owing to their chemical, structural, biological and fluorescent properties (Smith *et al.*, 1968 ▸; Sengupta & Kasha, 1979 ▸; Etter *et al.*, 1986 ▸; Klymchenko & Demchenko, 2002 ▸; Pivovarenko *et al.*, 2005 ▸; Choulier *et al.*, 2010 ▸). The phenomenon of dual fluorescence due to excited states intra­molecular proton transfer (ESIPT) has attracted much attention (Dick, 1987 ▸), as compounds exhibiting such properties can be used as fluorescent probes for sensing and imaging. The fluorescence of flavonols has been shown to be related to the angle between the 4*H*-chromene-4-one moiety and the attached benzene ring (Klymchenko *et al.* 2003 ▸). The effect of the intra­molecular hydrogen bond of flavonols, with an OH group in position 3, for the stabilization of the mol­ecular conformation is also important and this has been confirmed by theoretical calculations reported in a computational study on flavonoids (Aparicio, 2010 ▸). As a part of our search for new luminescent materials, we report herein on the synthesis and crystal structure of the title compound, the 4-*tert*-butyl­phenyl derivative of 3-hy­droxy­flavone.
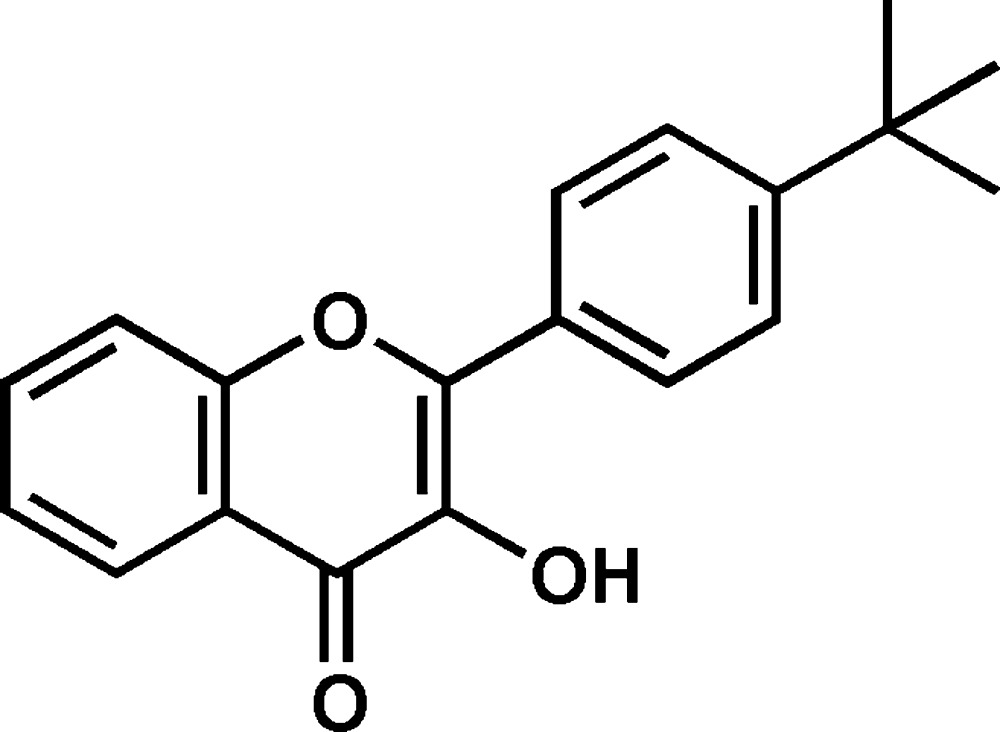


## Structural commentary   

**Figure 1:**
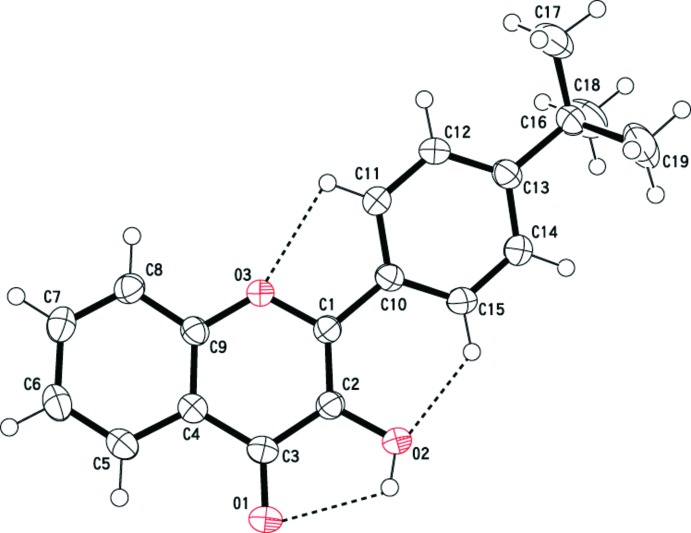
The mol­ecular structure of the title compound, showing the atom labelling. Displacement ellipsoids are drawn at the 50% probability level. Hydrogen bonds and short contacts are shown as dashed lines.

The mol­ecular structure of the title compound is illustrated in Fig. 1 ▸. The bond lengths are similar to those reported for other flavonols (Yoo *et al.*, 2014 ▸; Serdiuk *et al.*, 2013 ▸; Hino *et al.*, 2013 ▸, 2011 ▸; Wera, Pivovarenko *et al.*, 2011 ▸; Wera, Serdiuk *et al.*, 2011 ▸, Wera *et al.*, 2010 ▸). The mean plane of the 4*H*-chromene-4-one moiety (O3/C1–C9; r.m.s. deviation = 0.0191 Å) is twisted by 10.53 (8)° with respect to the benzene ring (C10–C16). This relative planarity typical of the structural features of flavonols is reinforced by two intra­molecular (C11—H11⋯O3 and C15—H15⋯O2) short contacts (Table 1 ▸ and Fig. 1 ▸). These intra­molecular contacts lead to the mol­ecular planarity and increase the torsional barrier, improving the π-delocalization from the 4*H*-chromene-4-one moiety toward the benzene ring. The mol­ecule also contains an intra­molecular O—H⋯O hydrogen bond (Table 1 ▸ and Fig. 1 ▸) with an *S*(5) ring motif.

## Supra­molecular features   

**Figure 2:**
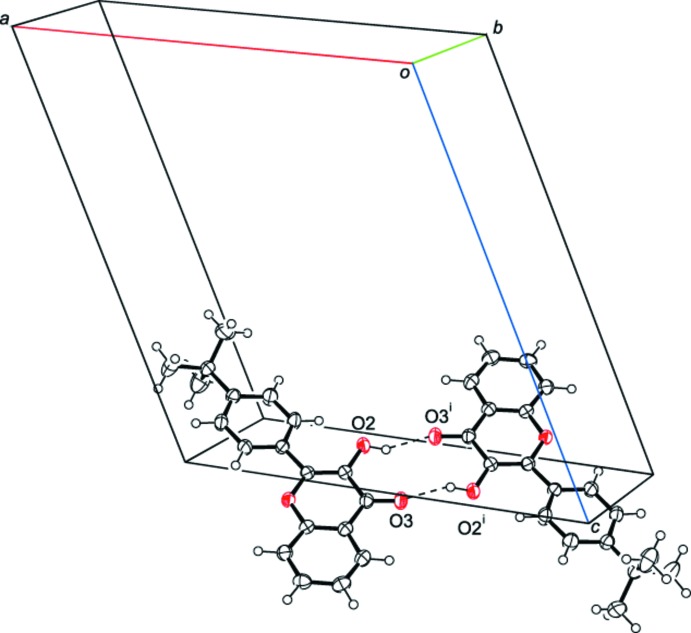
A view of the inversion dimer with an 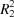
(10) ring motif. Dashed lines indicate hydrogen bonds. [Symmetry code: (i) −*x* + 1, −*y* + 1, −*z* + 2.]

**Figure 3:**
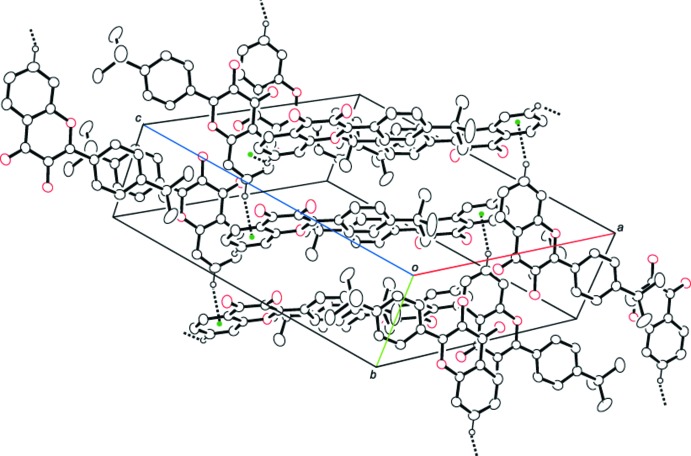
View of the crystal packing of the title compound. Dashed lines indicate the C—H⋯π inter­actions (ring centroids are shown as coloured spheres; see Table 1 ▸ for details). H atoms that do not participate in these inter­actions have been omitted for clarity.

In the crystal of the title compound, mol­ecules are linked *via* pairs of O—H⋯O hydrogen bonds, forming inversion dimers with an 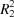
(10) ring motif (Table 1 ▸ and Fig. 2 ▸). The dimers are linked by C—H⋯π inter­actions between neighbouring mol­ecules, forming sheets parallel to (10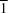); see Table 1 ▸ and Fig. 3 ▸.

## Database survey   

A search of the Cambridge Structural Database (Version 5.36, February 2015; Groom & Allen, 2014 ▸) for 3-hydoxyflavone gave 15 hits. These include 3-hy­droxy­flavone itself (DUMFAS; Etter *et al.*, 1986 ▸) and a number of *para*-substituted phenyl derivatives, such as the 4-amino­phenyl derivative (LUBBIV: Sun, 2015 ▸), two polymorphs of the 4-(di­methyl­amino)­phenyl derivative (BANJEH; BANJEH01: Hino *et al.*, 2011 ▸) and two polymorphs of the 4-(di­ethyl­amino)­phenyl derivative (CEZDOC; CEZDOC01: Hino *et al.*, 2013 ▸). Two polymorphs of the 4-hydroxphenyl derivative have also been reported (IJUCAS; Wera, Pivovarenko *et al.*, 2011 ▸; IKAHIM: Wera, Serdiuk *et al.*, 2011 ▸). Apart from 3-hy­droxy­flavone itself (DUMFAS) and the 4-amino­phenyl derivative (LUBBIV), in which the phenyl ring is inclined to the mean plane of the chromen-4-one moiety by 5.5 and 4.5°, respectively, this dihedral angle in the other compounds varies from 12.3 to 31.2°. Hence, in DUMFAS and LUBBIV there are also short intra­molecular C—H⋯O inter­actions, similar to those in the title compound. In the crystals of these two compounds, mol­ecules are also linked *via* O—H⋯O hydrogen bonds, but form chains. along [001] for DUMFAS and along [100] for LUBBIV, rather than inversion dimers as in the crystal of the title compound.

## Synthesis and crystallization   

The title compound was prepared by a modification of the procedure described by Qin *et al.* (2008 ▸). 2-Hy­droxy­aceto­phenone (1 mmol) was added to a suspension of the 4-*tert*-butyl­benzaldehyde (1 mmol) in ethanol (2 ml) and aqueous NaOH (6 *M*, 1 ml). The mixture was stirred at room temperature overnight. Then dilute acetic acid (30%) was added to the reaction mixture with stirring until the mixture was acidic and was cooled with an ice bath. The mixture was stirred for an additional 30 min at 273 K, and the solid precipitate obtained was collected by filtration. Hydrogen peroxide (30%, 2.6 mmol) was then added to an ice-cold suspension of the precipitate in ethanol (5 ml) and aqueous NaOH (2 *M*, 1 ml). The mixture was allowed to warm to room temperature and stirred for 4 h. The mixture was then acidified with dilute HCl (5%, 7 ml), and the precipitate formed was collected by filtration. Recrystallization from methanol gave yellow–green fluorescing crystals. Plate-like crystals suitable for X-ray diffraction analysis were obtained by slow evaporation of a solution in di­chloro­methane. ^1^H NMR (400MHz, DMSO-*d*_6_): δ 1.33 [*s*, 9H, C(CH_3_)_3_], 7.46 (*t*, 1H), 7.50 (*d*,2H), 7.79 (*dd*,2H), 8.13 (*dt*,3H), 9.50 (*s*,1H). ^13^C NMR (100MHz, DMSO-*d*_6_) δ 31.4, 35.1, 118.8, 121.8, 125.1, 125.8, 128.0, 129.0, 134.1, 139.3, 146.0, 153.2, 155.0, 173.3. Fluorescent emission maxima (CH_3_Cl, λ_ex_ = 365 nm): λ_em_ = 525 nm.

## Refinement   

Crystal data, data collection and structure refinement details are summarized in Table 2 ▸. The hydroxyl and C-bound H atoms were included in calculated positions and treated as riding atoms: O—H = 0.84 Å, C—H = 0.95–0.98 Å with *U*_iso_(H) = 1.5*U*_eq_(C,O) for the methyl and hydroxyl H atoms and 1.2*U*_eq_(C) for other H atoms.

## Supplementary Material

Crystal structure: contains datablock(s) global, I. DOI: 10.1107/S2056989015011482/su5150sup1.cif

Structure factors: contains datablock(s) I. DOI: 10.1107/S2056989015011482/su5150Isup2.hkl

Supporting information file. DOI: 10.1107/S2056989015011482/su5150Isup3.cml

CCDC reference: 1406583

Additional supporting information:  crystallographic information; 3D view; checkCIF report

## Figures and Tables

**Table 1:** Hydrogen-bond geometry (, ) *Cg* is the centroid of the C4C9 ring.

*D*H*A*	*D*H	H*A*	*D* *A*	*D*H*A*
O2H2O1	0.84	2.28	2.7262(14)	113
C11H11O3	0.95	2.32	2.6724(17)	101
C15H15O2	0.95	2.22	2.8508(18)	123
O2H2O1^i^	0.84	1.96	2.7104(14)	148
C7H7*Cg* ^ii^	0.95	2.59	3.407(10)	144

Symmetry codes: (i) 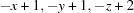; (ii) 
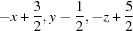
.

**Table 2:** Experimental details

Crystal data	
Chemical formula	C_19_H_18_O_3_
*M*_r_	294.33
Crystal system, space group	Monoclinic, *P*2_1_/*n*
Temperature (K)	200
*a*, *b*, *c* ()	15.9735(19), 6.1467(7), 16.963(2)
()	113.730(1)
*V* (^3^)	1524.7(3)
*Z*	4
Radiation type	Mo *K*
(mm^1^)	0.09
Crystal size (mm)	0.20 0.19 0.06

Data collection	
Diffractometer	Bruker APEXII CCD area detector
Absorption correction	Multi-scan (*SADABS*; Bruker 2014 ▸)
*T*_min_, *T*_max_	0.849, 0.928
No. of measured, independent and observed [*I* > 2(*I*)] reflections	15995, 3231, 2820
*R*_int_	0.026
(sin /)_max_ (^1^)	0.633

Refinement	
*R*[*F* ^2^ > 2(*F* ^2^)], *wR*(*F* ^2^), *S*	0.043, 0.127, 1.04
No. of reflections	3231
No. of parameters	203
H-atom treatment	H-atom parameters constrained
_max_, _min_ (e ^3^)	0.29, 0.23

Computer programs: *APEX2*, *SAINT* and *XPREP* (Bruker 2014 ▸), *SHELXS2014* (Sheldrick, 2008 ▸), *SHELXL2014* (Sheldrick, 2015 ▸), *ORTEP-3 for Windows* (Farrugia, 2012 ▸) and *PLATON* (Spek, 2009 ▸).

## supplementary crystallographic information

### Crystal data

**Table:**

C_19_H_18_O_3_	*F*(000) = 624
*M**_r_* = 294.33	*D*_x_ = 1.282 Mg m^−^^3^
Monoclinic, *P*2_1_/*n*	Mo *K*α radiation, λ = 0.71073 Å
*a* = 15.9735 (19) Å	Cell parameters from 7083 reflections
*b* = 6.1467 (7) Å	θ = 2.6–28.2°
*c* = 16.963 (2) Å	µ = 0.09 mm^−^^1^
β = 113.730 (1)°	*T* = 200 K
*V* = 1524.7 (3) Å^3^	Plate, yellow
*Z* = 4	0.20 × 0.19 × 0.06 mm

### Data collection

**Table:**

Bruker APEXII CCD area-detector diffractometer	3231 independent reflections
Radiation source: Bruker TXS fine-focus rotating anode	2820 reflections with *I* > 2σ(*I*)
Bruker Helios multilayer confocal mirror monochromator	*R*_int_ = 0.026
Detector resolution: 8.333 pixels mm^-1^	θ_max_ = 26.7°, θ_min_ = 1.5°
φ and ω scans	*h* = −20→20
Absorption correction: multi-scan (*SADABS*; Bruker 2014)	*k* = −7→7
*T*_min_ = 0.849, *T*_max_ = 0.928	*l* = −21→21
15995 measured reflections	

### Refinement

**Table:**

Refinement on *F*^2^	Primary atom site location: structure-invariant direct methods
Least-squares matrix: full	Secondary atom site location: difference Fourier map
*R*[*F*^2^ > 2σ(*F*^2^)] = 0.043	Hydrogen site location: inferred from neighbouring sites
*wR*(*F*^2^) = 0.127	H-atom parameters constrained
*S* = 1.04	*w* = 1/[σ^2^(*F*_o_^2^) + (0.0646*P*)^2^ + 0.6235*P*] where *P* = (*F*_o_^2^ + 2*F*_c_^2^)/3
3231 reflections	(Δ/σ)_max_ < 0.001
203 parameters	Δρ_max_ = 0.29 e Å^−^^3^
0 restraints	Δρ_min_ = −0.23 e Å^−^^3^

### Special details

**Table:**

Geometry. All esds (except the esd in the dihedral angle between two l.s. planes) are estimated using the full covariance matrix. The cell esds are taken into account individually in the estimation of esds in distances, angles and torsion angles; correlations between esds in cell parameters are only used when they are defined by crystal symmetry. An approximate (isotropic) treatment of cell esds is used for estimating esds involving l.s. planes. Least-squares planes (x,y,z in crystal coordinates) and deviations from them (* indicates atom used to define plane) 4.7085 (0.0060) x + 3.2010 (0.0016) y + 10.4295 (0.0043) z = 14.3715 (0.0031) * 0.0005 (0.0010) C1 * -0.0361 (0.0011) C2 * -0.0027 (0.0010) C3 * 0.0247 (0.0012) C4 * 0.0176 (0.0011) C5 * -0.0080 (0.0012) C6 * -0.0234 (0.0011) C7 * -0.0098 (0.0011) C8 * 0.0146 (0.0012) C9 * 0.0226 (0.0009) O3 Rms deviation of fitted atoms = 0.0191 6.5320 (0.0088) x + 2.3353 (0.0039) y + 10.0931 (0.0080) z = 15.2731 (0.0036) Angle to previous plane (with approximate esd) = 10.53 ( 0.08 ) * -0.0146 (0.0010) C10 * 0.0082 (0.0011) C11 * 0.0057 (0.0011) C12 * -0.0134 (0.0011) C13 * 0.0071 (0.0011) C14 * 0.0068 (0.0011) C15 Rms deviation of fitted atoms = 0.0099

### Fractional atomic coordinates and isotropic or equivalent isotropic displacement parameters (Å^2^)

**Table:**

	*x*	*y*	*z*	*U*_iso_*/*U*_eq_	
C1	0.71865 (9)	0.1616 (2)	1.00398 (8)	0.0265 (3)	
C2	0.64236 (9)	0.2637 (2)	1.00356 (8)	0.0276 (3)	
C3	0.59399 (9)	0.1820 (2)	1.05367 (8)	0.0281 (3)	
C4	0.62989 (9)	−0.0175 (2)	1.10134 (8)	0.0278 (3)	
C5	0.58831 (10)	−0.1190 (3)	1.15059 (9)	0.0346 (3)	
H5	0.5365	−0.0539	1.1552	0.041*	
C6	0.62214 (11)	−0.3121 (3)	1.19211 (10)	0.0381 (3)	
H6	0.5937	−0.3802	1.2252	0.046*	
C7	0.69847 (10)	−0.4080 (2)	1.18560 (9)	0.0351 (3)	
H7	0.7214	−0.5416	1.2143	0.042*	
C8	0.74088 (10)	−0.3116 (2)	1.13819 (9)	0.0324 (3)	
H8	0.7929	−0.3770	1.1341	0.039*	
C9	0.70584 (9)	−0.1159 (2)	1.09627 (8)	0.0271 (3)	
C10	0.77563 (9)	0.2243 (2)	0.95792 (8)	0.0274 (3)	
C11	0.83993 (10)	0.0782 (2)	0.95235 (9)	0.0340 (3)	
H11	0.8481	−0.0587	0.9805	0.041*	
C12	0.89195 (10)	0.1298 (3)	0.90651 (10)	0.0362 (3)	
H12	0.9349	0.0266	0.9037	0.043*	
C13	0.88329 (9)	0.3279 (2)	0.86439 (9)	0.0307 (3)	
C14	0.82088 (11)	0.4750 (3)	0.87277 (11)	0.0398 (4)	
H14	0.8143	0.6138	0.8463	0.048*	
C15	0.76821 (10)	0.4262 (2)	0.91811 (10)	0.0372 (3)	
H15	0.7265	0.5313	0.9222	0.045*	
C16	0.94007 (10)	0.3875 (3)	0.81313 (9)	0.0347 (3)	
C17	0.98436 (16)	0.1886 (3)	0.79140 (15)	0.0626 (6)	
H17A	1.0163	0.2333	0.7554	0.094*	
H17B	0.9370	0.0819	0.7602	0.094*	
H17C	1.0282	0.1231	0.8447	0.094*	
C18	1.01612 (14)	0.5414 (3)	0.86712 (15)	0.0606 (5)	
H18A	0.9894	0.6719	0.8808	0.091*	
H18B	1.0524	0.5828	0.8347	0.091*	
H18C	1.0555	0.4686	0.9206	0.091*	
C19	0.87973 (14)	0.4987 (5)	0.72920 (14)	0.0809 (8)	
H19A	0.8553	0.6342	0.7420	0.121*	
H19B	0.8290	0.4021	0.6958	0.121*	
H19C	0.9160	0.5316	0.6958	0.121*	
O1	0.52607 (7)	0.27779 (17)	1.05437 (7)	0.0367 (3)	
O2	0.60997 (7)	0.44503 (17)	0.95608 (7)	0.0359 (3)	
H2	0.5649	0.4923	0.9644	0.054*	
O3	0.74981 (6)	−0.02683 (15)	1.04986 (6)	0.0306 (2)	

### Atomic displacement parameters (Å^2^)

**Table:**

	*U*^11^	*U*^22^	*U*^33^	*U*^12^	*U*^13^	*U*^23^
C1	0.0279 (6)	0.0263 (6)	0.0258 (6)	0.0013 (5)	0.0114 (5)	0.0006 (5)
C2	0.0276 (6)	0.0279 (7)	0.0272 (6)	0.0021 (5)	0.0108 (5)	0.0009 (5)
C3	0.0250 (6)	0.0311 (7)	0.0282 (6)	−0.0002 (5)	0.0107 (5)	−0.0031 (5)
C4	0.0279 (6)	0.0288 (7)	0.0268 (6)	−0.0025 (5)	0.0112 (5)	−0.0027 (5)
C5	0.0328 (7)	0.0386 (8)	0.0373 (7)	−0.0024 (6)	0.0193 (6)	−0.0004 (6)
C6	0.0441 (8)	0.0371 (8)	0.0385 (8)	−0.0071 (6)	0.0222 (7)	0.0025 (6)
C7	0.0447 (8)	0.0273 (7)	0.0319 (7)	−0.0016 (6)	0.0141 (6)	0.0016 (6)
C8	0.0372 (7)	0.0287 (7)	0.0326 (7)	0.0032 (6)	0.0154 (6)	0.0001 (5)
C9	0.0297 (6)	0.0283 (7)	0.0251 (6)	−0.0017 (5)	0.0128 (5)	−0.0017 (5)
C10	0.0270 (6)	0.0298 (7)	0.0265 (6)	0.0004 (5)	0.0120 (5)	−0.0009 (5)
C11	0.0368 (7)	0.0314 (7)	0.0384 (7)	0.0077 (6)	0.0199 (6)	0.0082 (6)
C12	0.0359 (7)	0.0367 (8)	0.0432 (8)	0.0100 (6)	0.0235 (7)	0.0067 (6)
C13	0.0281 (6)	0.0354 (7)	0.0307 (7)	−0.0009 (5)	0.0140 (5)	0.0006 (5)
C14	0.0456 (9)	0.0312 (8)	0.0525 (9)	0.0067 (6)	0.0300 (8)	0.0113 (7)
C15	0.0411 (8)	0.0300 (7)	0.0508 (9)	0.0080 (6)	0.0291 (7)	0.0060 (6)
C16	0.0321 (7)	0.0395 (8)	0.0376 (7)	0.0019 (6)	0.0195 (6)	0.0062 (6)
C17	0.0852 (14)	0.0522 (11)	0.0822 (14)	0.0007 (10)	0.0668 (13)	−0.0014 (10)
C18	0.0534 (11)	0.0642 (12)	0.0782 (13)	−0.0170 (9)	0.0411 (10)	−0.0115 (10)
C19	0.0521 (11)	0.144 (2)	0.0597 (12)	0.0286 (13)	0.0358 (10)	0.0514 (14)
O1	0.0309 (5)	0.0397 (6)	0.0455 (6)	0.0085 (4)	0.0214 (5)	0.0056 (5)
O2	0.0327 (5)	0.0376 (6)	0.0433 (6)	0.0125 (4)	0.0215 (5)	0.0132 (5)
O3	0.0333 (5)	0.0289 (5)	0.0352 (5)	0.0068 (4)	0.0198 (4)	0.0062 (4)

### Geometric parameters (Å, º)

**Table:**

C1—C2	1.3682 (18)	C11—H11	0.9500
C1—O3	1.3723 (16)	C12—C13	1.390 (2)
C1—C10	1.4695 (18)	C12—H12	0.9500
C2—O2	1.3502 (16)	C13—C14	1.395 (2)
C2—C3	1.4493 (18)	C13—C16	1.5323 (18)
C3—O1	1.2386 (16)	C14—C15	1.382 (2)
C3—C4	1.4537 (19)	C14—H14	0.9500
C4—C9	1.3888 (19)	C15—H15	0.9500
C4—C5	1.4056 (19)	C16—C18	1.521 (2)
C5—C6	1.375 (2)	C16—C19	1.523 (2)
C5—H5	0.9500	C16—C17	1.530 (2)
C6—C7	1.398 (2)	C17—H17A	0.9800
C6—H6	0.9500	C17—H17B	0.9800
C7—C8	1.376 (2)	C17—H17C	0.9800
C7—H7	0.9500	C18—H18A	0.9800
C8—C9	1.3952 (19)	C18—H18B	0.9800
C8—H8	0.9500	C18—H18C	0.9800
C9—O3	1.3629 (16)	C19—H19A	0.9800
C10—C15	1.395 (2)	C19—H19B	0.9800
C10—C11	1.3956 (19)	C19—H19C	0.9800
C11—C12	1.3840 (19)	O2—H2	0.8400
			
C2—C1—O3	120.60 (12)	C12—C13—C14	116.28 (13)
C2—C1—C10	128.16 (12)	C12—C13—C16	122.81 (13)
O3—C1—C10	111.22 (11)	C14—C13—C16	120.89 (13)
O2—C2—C1	120.56 (12)	C15—C14—C13	122.42 (14)
O2—C2—C3	117.97 (11)	C15—C14—H14	118.8
C1—C2—C3	121.47 (12)	C13—C14—H14	118.8
O1—C3—C2	121.12 (13)	C14—C15—C10	120.67 (13)
O1—C3—C4	123.13 (12)	C14—C15—H15	119.7
C2—C3—C4	115.74 (11)	C10—C15—H15	119.7
C9—C4—C5	118.49 (13)	C18—C16—C19	109.58 (17)
C9—C4—C3	119.46 (12)	C18—C16—C17	107.91 (15)
C5—C4—C3	122.02 (12)	C19—C16—C17	108.29 (17)
C6—C5—C4	120.36 (13)	C18—C16—C13	108.58 (13)
C6—C5—H5	119.8	C19—C16—C13	109.96 (12)
C4—C5—H5	119.8	C17—C16—C13	112.48 (13)
C5—C6—C7	119.94 (13)	C16—C17—H17A	109.5
C5—C6—H6	120.0	C16—C17—H17B	109.5
C7—C6—H6	120.0	H17A—C17—H17B	109.5
C8—C7—C6	120.97 (14)	C16—C17—H17C	109.5
C8—C7—H7	119.5	H17A—C17—H17C	109.5
C6—C7—H7	119.5	H17B—C17—H17C	109.5
C7—C8—C9	118.55 (13)	C16—C18—H18A	109.5
C7—C8—H8	120.7	C16—C18—H18B	109.5
C9—C8—H8	120.7	H18A—C18—H18B	109.5
O3—C9—C4	121.80 (12)	C16—C18—H18C	109.5
O3—C9—C8	116.51 (12)	H18A—C18—H18C	109.5
C4—C9—C8	121.69 (12)	H18B—C18—H18C	109.5
C15—C10—C11	117.45 (12)	C16—C19—H19A	109.5
C15—C10—C1	122.70 (12)	C16—C19—H19B	109.5
C11—C10—C1	119.84 (12)	H19A—C19—H19B	109.5
C12—C11—C10	121.02 (13)	C16—C19—H19C	109.5
C12—C11—H11	119.5	H19A—C19—H19C	109.5
C10—C11—H11	119.5	H19B—C19—H19C	109.5
C11—C12—C13	122.10 (13)	C2—O2—H2	109.5
C11—C12—H12	119.0	C9—O3—C1	120.86 (10)
C13—C12—H12	119.0		
			
O3—C1—C2—O2	−177.75 (11)	O3—C1—C10—C15	−169.26 (13)
C10—C1—C2—O2	0.6 (2)	C2—C1—C10—C11	−167.35 (14)
O3—C1—C2—C3	2.3 (2)	O3—C1—C10—C11	11.16 (18)
C10—C1—C2—C3	−179.29 (12)	C15—C10—C11—C12	−2.2 (2)
O2—C2—C3—O1	−1.4 (2)	C1—C10—C11—C12	177.44 (13)
C1—C2—C3—O1	178.52 (13)	C10—C11—C12—C13	0.3 (2)
O2—C2—C3—C4	178.03 (11)	C11—C12—C13—C14	1.7 (2)
C1—C2—C3—C4	−2.04 (19)	C11—C12—C13—C16	−179.81 (14)
O1—C3—C4—C9	179.28 (13)	C12—C13—C14—C15	−1.8 (2)
C2—C3—C4—C9	−0.15 (18)	C16—C13—C14—C15	179.63 (14)
O1—C3—C4—C5	1.4 (2)	C13—C14—C15—C10	0.0 (3)
C2—C3—C4—C5	−178.04 (12)	C11—C10—C15—C14	2.0 (2)
C9—C4—C5—C6	−0.4 (2)	C1—C10—C15—C14	−177.57 (14)
C3—C4—C5—C6	177.48 (13)	C12—C13—C16—C18	−102.50 (18)
C4—C5—C6—C7	0.2 (2)	C14—C13—C16—C18	75.92 (19)
C5—C6—C7—C8	0.2 (2)	C12—C13—C16—C19	137.61 (19)
C6—C7—C8—C9	−0.2 (2)	C14—C13—C16—C19	−44.0 (2)
C5—C4—C9—O3	−179.91 (12)	C12—C13—C16—C17	16.8 (2)
C3—C4—C9—O3	2.13 (19)	C14—C13—C16—C17	−164.73 (16)
C5—C4—C9—C8	0.4 (2)	C4—C9—O3—C1	−1.97 (19)
C3—C4—C9—C8	−177.60 (12)	C8—C9—O3—C1	177.77 (11)
C7—C8—C9—O3	−179.77 (12)	C2—C1—O3—C9	−0.30 (19)
C7—C8—C9—C4	0.0 (2)	C10—C1—O3—C9	−178.94 (11)
C2—C1—C10—C15	12.2 (2)		

### Hydrogen-bond geometry (Å, º)

Cg is the centroid of the C4–C9 ring.

**Table:**

*D*—H···*A*	*D*—H	H···*A*	*D*···*A*	*D*—H···*A*
O2—H2···O1	0.84	2.28	2.7262 (14)	113
C11—H11···O3	0.95	2.32	2.6724 (17)	101
C15—H15···O2	0.95	2.22	2.8508 (18)	123
O2—H2···O1^i^	0.84	1.96	2.7104 (14)	148
C7—H7···*Cg*^ii^	0.95	2.59	3.407 (10)	144

Symmetry codes: (i) −*x*+1, −*y*+1, −*z*+2; (ii) −*x*+3/2, *y*−1/2, −*z*+5/2.
